# Racial Disparities in Homicide Victimisation Rates: How to Improve Transparency by the Office of National Statistics in England and Wales

**DOI:** 10.1007/s41887-020-00055-y

**Published:** 2020-11-10

**Authors:** Sumit Kumar, Lawrence W. Sherman, Heather Strang

**Affiliations:** Jerry Lee Centre for Experimental Criminology, Institute of Criminology, University of Cambridge and Cambridge Centre for Evidence-Based Policing Ltd., Cambridge, UK

**Keywords:** Homicide victimisation, Rates versus counts, Racial disparity, Crime statistics, Policing disparity

## Abstract

**Research Question:**

How much racial disparity in trends of homicide victimisation rates in England and Wales is obscured by the failure of official statistics to report rates of death per 100,000 people at risk?

**Data:**

We collected two decades of homicide victimisation counts in England and Wales, as broken out for each racial group identified by the Office of National Statistics. We also collected the estimated population size of those groups from the 2001 and 2011 Census.

**Methods:**

We divided the number of homicides in each racial category by the estimated population size of that category, by year, for 20 years, and plotted their relationships.

**Findings:**

While White homicide victimisation rates remained low and stable from 2000 through 2019, Black homicide victimisation ranged from 200 to 800% higher than that for the White population during that time period, at an average of 5.6 times higher for Blacks. While Black victimisation dropped by 69% from 2001 to 2012, it almost doubled (79% increase) from 2013 to 2019, rising seven times faster than the White victimisation rate. Asian rates remained stable at about twice as high as White rates. For persons aged 16 to 24, the most recent homicide rate was 24 times higher for Blacks than for Whites.

**Conclusion:**

None of these rates per 100,000 or ratios has been reported by the Office of National Statistics. If future ONS reporting of homicide rates would include relevant denominators with raw numerators, public understanding of racial disparities in “over-policing” could be informed by potential “under-policing” relative to racial inequalities in homicide risk.

## Introduction

The issue of systemic racism in policing is most often framed as one of greater intrusions by police upon minorities than upon Whites, under similar circumstances. Far more damaging in terms of the life expectancy of racial minorities, however, is the widespread systemic racial difference in homicide victimisation rates. When members of one group are far more likely to be murdered than members of other groups, it should be of equal concern as other disparities in mortality, such as from COVID-19, for which official statistics in England and Wales report death rates per 100,000 for Whites vs ethnic minorities (White & Nafilyan 2020). That concern cannot be identified for homicide, however, if the rates themselves are not calculated and reported as a routine part of official statistics.

Official reporting of racially disaggregated homicide victimisation rates has long been a standard practice in the USA (Reiss & Roth 1993), Australia (Strang, 1991), and other economically advanced nations. Yet as far as we can tell, homicide victimisation rates have never been reported by race in England and Wales in the twenty-first century, either by the Office of National Statistics or by the Home Office before the ONS took over the duty of reporting crime statistics.

It is only the lack of adequate governmental transparency on risks of murder victimisation by race that makes it necessary to publish this research note. To be precise, what the crime statistics for England and Wales fail to report by race is the simple division of the number of homicides in each racial group by the estimated number of people of that group residing in England and Wales. This paper takes that simple step, in order to show important facts that have heretofore been largely invisible.

It is an elementary principle of statistics that comparisons of raw numbers of rare events across groups of different sizes cannot be interpreted meaningfully. It is only by applying the basic idea of fractions that such comparisons can be interpreted. Whatever the numerator may be, it takes a denominator to make it useful in comparing two groups. That is why, homicide comparisons across nations are expressed in *rates* of homicide per 100,000 people, rather than *counts* of people killed in nations of vastly different population sizes.

While the 2020 Office of National Statistics (2020) annual report on homicide in England and Wales reports the *counts* of homicide victims by ethnic classification, it does so without computing race-specific rates per 100,000. The report even offers, but does not deliver, these comparisons in section on “Which groups of people were most likely to be victims of homicide?” Yet the question is largely unanswered, limiting computation of homicides per 100,000s to differences across age groups, aggregated across all races. It does not give the reader any assistance in analysing the relative differences in rates of murder across different kinds of people or even different kinds of differences.

Sadly, the lack of comparable rates is a common problem in reporting important numbers. For well over 6 months of the COVID-19 pandemic, BBC News reported daily infection counts of the *number* of infections detected in different locations, rather than a standardised rate per 100,000 people at risk of infection. The fact that the problem has now been corrected for COVID-19 offers a precedent for ONS to offer more differentiation in homicides per 100,000 across ethnicity, age, areas of the country, and combinations of this dimensions. This article illustrates what can be done by simple arithmetic with the published data from the last two decades.

Other important questions, such as the years of potential life lost (YPLL), could also be considered for racial disparity, as well as disparities over time and across communities (see Reiss and Roth, eds. 1993). Such analyses can acquire greater investment if they can build on the foundation of racial disaggregation of homicide rates per 100,000 people.

## Research question

Our research question is *how much racial disparity in trends of homicide victimisation rates in England and Wales is obscured by the failure of official statistics to report rates of death per 100,000 people at risk?* We focus this question at the national level, but it could be equally applied to every one of the 43 territorial police forces in England and Wales.

## Data

We collected two decades of homicide victimisation counts in England and Wales, as broken out by either the Home Office or the Office of National Statistics, for each racial group identified in the decennial Census reports by the Office of National Statistics. We also collected the estimated population size of those groups from the 2001 and 2011 Census. To the extent possible, we tried to match the definitions of ethnic groups between the Census categories and the homicide categories.

Our challenge was that the classification of Asian ethnicity in the Census, and ONS data of homicide victims was different. In the ONS homicide victimisation data, Asian included only victims from the Indian subcontinent. In the census data, Asian included people from all of the continent of Asia. Our least-worst solution to this challenge was to match the definition across the two datasets by using the following classification: White (White British, White Irish, and White Gypsy and White other); Black (Black African, Black Caribbean, and Black other); Asian, Indian subcontinent (Pakistan, India, Bangladesh, Sri Lankan); and Other (Arabs; Chinese; Asian [other]; mixed; any other). Using these definitions appears to offer the most precise common boundaries possible around numerators and denominators.

## Methods

We divided the number of homicides in each racial category by the estimated population size of that category, by year, for 20 years, and plotted their relationships. We did not estimate changing sizes of the population of each ethnic group considered. We applied the 2001 denominator up through 2010–2011 and then applied the 2011 denominator for all years thereafter.

For one analysis only, we computed the rates of homicide for persons age 16–24 based solely on the 2011 Census count of persons of that age in each ethnic group (actually using the population data for person age 15–24, which were readily available by ethnicity from the Census). We were able to get the data by combination of age and ethnicity only from 2008 to 2018, with the 2011 Census data falling near the middle of that time-span. Thus, the statistics for calculating the rate for Black and White victims per 100,000 people in this age group are based on just the 2011 Census.

## Findings

We find racial differences in homicide victimisation rates between Blacks and Whites to be both substantial and dynamic. Death rates are consistently higher for Blacks than for Whites and Asians; Asian death rates are about twice as high as for Whites. The greatest difference visible from reported ONS data is among persons aged 16–24, where the most recent statistics show Black death rates to be 24 times higher per 100,000 than for Whites.

Table 1 shows the raw data from homicide counts by ethnic group on which we base the analysis. It demonstrates the important point that by far, the largest number of homicide victims in each year is White. This means that White deaths can always dominate the news and that by raw numbers alone may obscure the vast differences in underlying risk by race. Table 1 also shows that 2.5% had no record of victim ethnicity. That statistic, while small, is a finding that reflects violations of crime reporting requirements by police or others generating the initial crime report.

**Table 1:** Homicide victims by ethnicity in England and Wales

Year	Black	Asian	White	Other	Total*
2000/2001	67	49	552	25	693
2001/2002	106	62	588	24	780
2002/2003	88	46	577	35	746
2003/2004	76	62	563	18	719
2004/2005	90	48	565	32	735
2005/2006	70	53	492	15	630
2006/2007	92	70	489	27	678
2007/2008	89	54	543	24	710
2008/2009	87	47	473	24	631
2009/2010	61	51	448	21	581
2010/2011	65	62	464	19	610
2011/2012	66	44	387	16	513
2012/2013	53	32	433	12	530
2013/2014	58	58	371	12	499
2014/2015	58	32	384	16	490
2015/2016	64	35	425	17	541
2016/2017	86	42	420	12	560
2017/2018	95	51	477	20	643
2018/2019	97	42	475	24	638

*Excluding cases with race of victims unknown/not classified, 2.5% of total homicides (2009–2019)

(Source: Office of National Statistics)

Table 2 reports the denominators and definitions we use for the ethnic classifications with which we calculate the death rates per 100,000 for each group.

**Table 2:** Ethnically identified population in England and Wales

		Population (millions)	
2011	Per cent	Total	Age 15–24
England and Wales	100%	56.1	7.3
White	86%	48.2	6.0
Black	3.30%	1.9	0.28
Asian	5.30%	2.9	--
Other	5.40%	3.0	--
2001	Per cent	Total	
England and Wales	100%	52.4	
White	91.3%	47.8	
Black	2.20%	1.2	
Asian	4.40%	2.3	
Other	2.10%	1.1	

White: White British, White Irish, and White Gypsy and White other

Black: Black African, Black Caribbean, and Black other

Asian: Indian subcontinent (Pakistan, India, Bangladesh, Sri Lanka)

Other: Arabs; Chinese; Asian [other]; mixed; any other

(Source: Office of National Statistics)

**Fig. 1:**
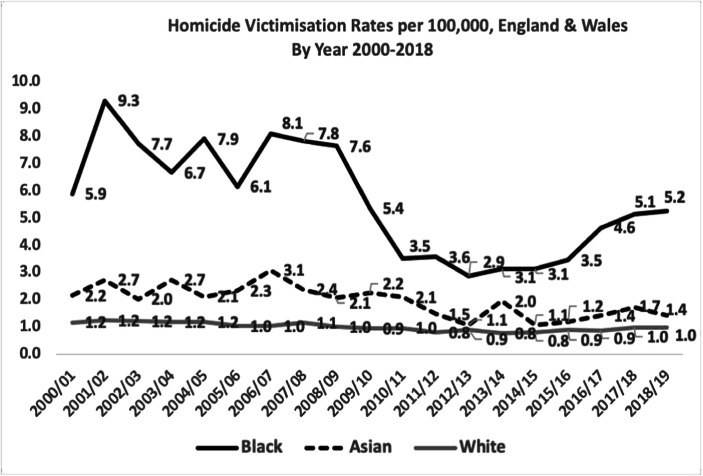
Homicide victimisation rates per 100,000, England and Wales by year 2000–2018

Table 3 shows the annual homicide victimisation rates per 100,000 persons at risk in each ethnic classification for the first 19 years of the twenty-first century. These statistics are the underlying data for the comparison of trends in Fig. 1.

**Table 3:** Homicide victimisation rate per 100,000 population by ethnicity in England and Wales

Year	Black	Asian	White	Other
2000/2001	5.9	2.2	1.2	2.3
2001/2002	9.3	2.7	1.2	2.2
2002/2003	7.7	2.0	1.2	3.2
2003/2004	6.7	2.7	1.2	1.6
2004/2005	7.9	2.1	1.2	2.9
2005/2006	6.1	2.3	1.0	1.4
2006/2007	8.1	3.1	1.0	2.4
2007/2008	7.8	2.4	1.1	2.2
2008/2009	7.6	2.1	1.0	2.2
2009/2010	5.4	2.2	0.9	1.9
2010/2011	3.5	2.1	1.0	0.7
2011/2012	3.6	1.5	0.8	0.5
2012/2013	2.9	1.1	0.9	0.4
2013/2014	3.1	2.0	0.8	0.4
2014/2015	3.1	1.1	0.8	0.5
2015/2016	3.5	1.2	0.9	0.6
2016/2017	4.6	1.4	0.9	0.4
2017/2018	5.1	1.7	1.0	0.7
2018/2019	5.2	1.4	1.0	0.8
Mean	5.6	2.0	1.0	1.4

Figure 1 displays three trend lines across the first 19 years of the twenty-first century, one for each specific ethnic group. It shows that the Black victimisation rate has been consistently the highest throughout this century and by a substantial margin. The mean rate for Blacks is 5.6 times higher than for Whites, while the most recent rate is 5.2.

Perhaps the most important pattern in Fig. 1 is the massive drop in the Black homicide victimisation rate from 8.1 in 2006–2007 to 2.9 in 2012–2013. Despite that 64% decline in the Black death rate, it was soon followed by an increase from 2015/2016 to 5.2 per 100,000 in 2018/2019—an increase of 79%. At the same time, the White death rate increased by only 11%, from 0.9 to 1.0 per 100,000. The Black homicide victimisation rate therefore rose seven times faster than the White homicide death rate.

Figure 1 also shows how Asian rates remained higher than the White rates but were far more stable than the Black homicide victimisation rates. At no time were they as much higher than the White rates as the Black rates were.

Table 4 displays the death rates per 100,000 by race and year for Blacks and Whites age 16–24, which is a point in the life span when homicide risk is often the highest (Reiss and Roth 1993; Strang 1991). The concentration of risk in those years, by definition, produces far higher rates of victimisation per 100,000.

**Table 4:** Homicide victimisation rate per 100,000 for ages 16–24 Black vs. White

	Black homicide victims (age 16–24)		White homicide victims (age 16–24)		
	Total	Rate (per 100,000)	Total	Rate (per 100,000)	Ratio (Black: White)
2008/2009	34	11.9	79	1.3	9:1
2009/2010	23	8.1	65	1.1	7:1
2010/2011	22	7.7	63	1.0	8:1
2011/2012	27	9.5	53	0.9	10:1
2012/2013	17	6.0	60	1.0	6:1
2013/2014	27	9.5	53	0.9	10:1
2014/2015	24	8.4	52	0.9	9:1
2015/2016	30	10.5	52	0.9	12:1
2016/2017	36	12.6	96	1.6	8:1
2017/2018	46	16.1	76	1.3	12:1
2018/2019	47	16.5	44	0.7	24:1
Mean		10.6		1.0	11:1

Table 4 shows far greater racial disparity between Blacks and Whites in this age group than in the entire population. That difference does not occur by definition but by substantive differences in risks. While overall White rates may be reduced by a larger proportion of Whites than of Blacks in older age groups with lower homicide rates, that complexity is absent from Table 4. What that table displays is a more closely matched like-for-like comparison: given people in the same age group in England and Wales, as between Whites and Blacks, who is more likely to be murdered? The answer is that Blacks are almost 11 (10.6) times more likely to be murdered than Whites.

These differences do not control for sex. ONS data do not include even raw numbers of homicide deaths by sex, age, and race combined. If they did, the concentration of murder victimisation among males would likely make these differences even more pronounced, as they are in the USA (Reiss & Roth 1993: 64).

**Fig. 2:**
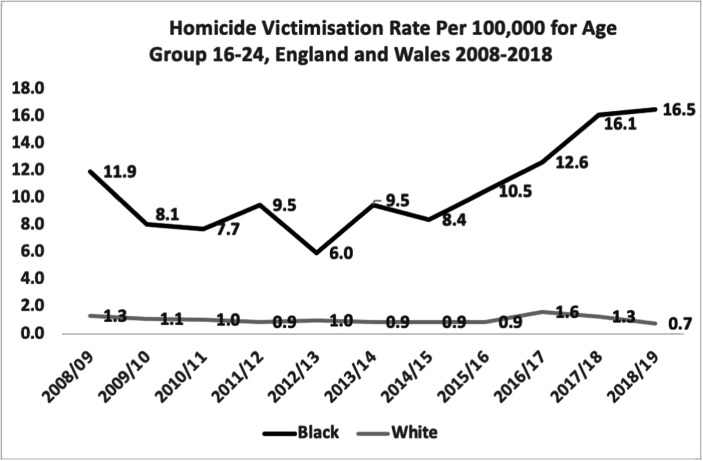
Homicide victimisation rate per 100,000 for age group 16–24, England and Wales 2008–2018

Figure 2 shows very little correlation between the death rate trends of young Whites and young Blacks. This is especially noticeable in the last 3 years of the time series, when the White homicide rate 16–24 dropped by 57%, while the Black homicide victimisations per 100,000 age 16–24 increased by 31%. Whatever the causes of the changes in each group, they appear to be largely independent of each other.

## Conclusions

These findings demonstrate the importance of making fair comparisons of risks based on fully calculated rates. While the principle is widely accepted, it is widely violated in practice. Evidence-based policing requires reliable evidence, including rates of risk calculated in the same manner for all racial groups. Only rates per 100,000 for any relevant numerator provide that kind of fair comparison.

What these rates show in substantive terms is a substantial racial inequality in risks of being murdered in England and Wales. While this article demonstrates inequality at a national level, it may be far greater—or even lower—in local areas, including police forces and their basic command units. While the ONS is not funded to do such calculations, it is arguably essential that territorial police forces do so themselves.

What the ONS is funded to do is to publish meaningful statistics. The article shows how vast the racial differences are in England and Wales in homicide victimisation rates per 100,000. These findings should provide all the evidence needed to expand annual (and retrospective) reporting of homicides to calculate race-specific and race-age-sex-specific rates of homicide victimisation per 100,000.

These calculations do not require an increase in anyone’s budget. They may, however, empower police to target racial disparities in a more precise way and to promote a more fact-informed dialogue with their many publics (Bottoms and Tankebe 2012). These facts may even help to save lives.

## References

[CR1] Bottoms, A., & Tankebe, J. (2012). Beyond procedural justice: A dialogic approach to legitimacy in criminal justice. *The Journal of Criminal Law and Criminology*, 119–170.

[CR2] Reiss AJ, Roth JA (1993). Understanding and preventing violence: Panel on the understanding and controlof violence behavior.

[CR3] Strang H (1991). Homicides in Australia 1990–91.

[CR4] White C, Nafilyan V (2020). Coronavirus (COVID-19) related deaths by ethnic group, England and Wales: 2 March 2020 to 10 April 2020.

